# Dopaminergic REST is implicated in the tamoxifen-induced neuroprotection against manganese toxicity in female mice

**DOI:** 10.3389/fphar.2025.1627400

**Published:** 2025-10-08

**Authors:** Edward Pajarillo, Alexis Digman, Itunu Ajayi, Sanghoon Kim, Deok-Soo Son, Michael Aschner, Eunsook Lee

**Affiliations:** ^1^ Department of Pharmaceutical Science, College of Pharmacy and Pharmaceutical Sciences, Florida A&M University, Tallahassee, FL, United States; ^2^ Department of Biochemistry, Cancer Biology, Neuroscience and Pharmacology, Meharry Medical College, Nashville, TN, United States; ^3^ Department of Molecular Pharmacology, Albert Einstein College of Medicine, New York, NY, United States

**Keywords:** epigenetic modulation, gene expression, histone acetylation, manganese, NRSF/REST, SERM, tamoxifen

## Abstract

**Introduction:**

Chronic manganese (Mn) overexposure causes manganism, a Parkinson’s disease-like neurological disorder, due to its preferential accumulation in the basal ganglia. Tamoxifen (TX), a selective estrogen receptor modulator (SERM), afforded neuroprotection against Mn toxicity, and RE1-silencing transcription factor (REST) in dopaminergic neurons mitigated Mn-induced neurotoxicity.

**Methods:**

This study investigated whether dopaminergic REST played a role in TX’s protection against Mn toxicity in the nigrostriatal regions using wild-type (WT) and dopaminergic REST-deleted (REST cKO) female mice. Behavioral studies, including open-field, rotarod, and novel object recognition tests, were conducted with molecular biology assays.

**Results:**

TX mitigated Mn-induced deficits in several motor functions and cognition, as well as dopaminergic neuronal injury, parallel with the attenuation of Mn-decreased tyrosine hydroxylase (TH) and Mn-increased proapoptotic Bax in REST cKO mice. However, several Mn-dysregulated genes associated with oxidative stress and mitochondrial function, including catalase, superoxide dismutase 2 (SOD2), and optic atrophy 1 (OPA1), were attenuated by TX only in WT, but not in REST cKO. At the epigenetic levels, TX attenuated Mn-reduced acetylation of H3K27 in both WT and REST cKO, but Mn-decreased H3K27ac interaction with promoters of catalase, SOD2, and OPA1 was attenuated by TX only in WT, not REST cKO. TX attenuated Mn-decreased estrogen receptor (ER)-α and ER-β protein levels in both WT and REST cKO mice.

**Discussion:**

Our findings suggest that TX significantly attenuated Mn-induced TH reduction and behavioral deficits in REST cKO, not to the levels of its protection in WT, since several genes involved in TX-induced protective pathways required dopaminergic REST. Taken together, while TX has some REST-independent protective effects, dopaminergic REST is critical for full neuroprotection.

## 1 Introduction

Chronic exposure to elevated levels of manganese (Mn) causes a neurological condition known as manganism, presenting symptoms that resemble those of Parkinson’s disease (PD), including tremors, dystonia, abnormal gait, and bradykinesia (Blomlie et al., 2020; Balachandran et al., 2020), as well as cognitive impairments (Banta and Markesbery, 1977; Liang et al., 2015).

Despite extensive research, no effective treatments currently exist to reverse or cure manganism. Given the growing public health concern posed by Mn exposure, the development of targeted therapeutic strategies is urgently needed. While several natural and synthetic compounds have shown promise in various animal models [for review, see Pajarillo et al. (2022b)], none have progressed to clinical use, underscoring the critical need to identify molecular pathways underlying Mn-induced toxicity.

Although the precise cellular and molecular mechanisms underlying Mn-induced neurotoxicity remain unclear, Mn has been shown to disrupt key biological functions in different neural cell types [for review, see Pajarillo et al. (2022b)]. Particularly, dopaminergic neuronal dysfunction, such as reduced dopamine synthesis and impaired neurotransmission, is primarily implicated in Mn-induced neurotoxicity linked to motor deficits. Mn decreased the expression of tyrosine hydroxylase (TH), the rate-limiting enzyme in dopamine synthesis, while reducing TH function by inhibiting TH phosphorylation (Kumasaka et al., 2017; Zhang et al., 2011; Pajarillo et al., 2020b). Mn also induces mitochondrial dysfunction, oxidative stress, and apoptosis in various neural cell types, which can lead to neuronal damage [for review, see Harischandra et al. (2019)]. Mn increases oxidative stress, at least in part, by inhibiting the expression of reactive oxygen species-scavenging genes (Yang et al., 2022). Mn also promotes apoptotic cell death by increasing proapoptotic genes, while decreasing antiapoptotic genes in neuronal cells and mice. Consequently, understanding the mechanisms involved in oxidative stress, mitochondrial dysfunction, and dopaminergic dysregulation is crucial for developing therapies to mitigate Mn-induced neurotoxicity.

Repressor element-1 silencing transcription factor (REST), also known as neuron restrictive silencer factor (NRSF), is a Kruppel-type zinc finger transcription factor (Chong et al., 1995; Schoenherr and Anderson, 1995). It plays a role in embryogenesis, neuronal differentiation, synaptogenesis, and axonal formation (Sun et al., 2005; Jorgensen et al., 2009; Paquette et al., 2000). Initially discovered as a transcriptional repressor of neuronal genes in non-neuronal cells (Jones and Meech, 1999; Schoenherr and Anderson, 1995) REST is also a transcriptional activator of genes, such as TH (Pajarillo et al., 2020b), excitatory amino acid transporter 2 (EAAT2) (Pajarillo et al., 2021), glutamate receptor 2 (GluR2) (Brene et al., 2000), and dynamin I (Yoo et al., 2001). As a transcription factor, REST regulates gene expression by recruiting various co-regulators, such as histone acetyltransferases (HAT), histone deacetylases (HDAC), CoREST, mSin3a, Tet methylcytosine dioxygenase 3, and nuclear receptor-binding SET domain 3 (Hwang and Zukin, 2018; Perera et al., 2015). Studies have shown that its ability to influence gene activation or repression also depends on the specific RE1-binding sequence motifs in the promoter regions of target genes (Bruce et al., 2009), indicating further research is required to understand REST’s regulatory mechanisms.

Dysregulation of REST has been implicated in several neurodegenerative diseases, including Alzheimer’s disease (AD), PD, ischemia, and Huntington’s disease (Ryan et al., 2021; Lu et al., 2014; Kawamura et al., 2019; Jiang et al., 2024; Baldelli and Meldolesi, 2015). In healthy aging brains, REST expression is high, but its low expression is found in the brains of AD patients (Lu et al., 2014). At the molecular level, REST exerts protection by activating antioxidant and antiapoptotic genes while reducing proapoptotic genes (Lu et al., 2014; Pajarillo et al., 2020b). In addition, dysregulation of REST is associated with impairment of autophagy, leading to α-synuclein accumulation in PD animal models (Rocchi et al., 2021; Ryan et al., 2021). Mn reduced REST expression in the midbrain and striatum of mice, parallel with Mn-induced oxidative stress, mitochondrial dysfunction, inflammation, and excitotoxicity (Pajarillo et al., 2024; Pajarillo et al., 2022a). Restoration of REST exerted protection against Mn-induced dopaminergic toxicity, partly by attenuating Mn-induced oxidative stress and autophagy impairment, resulting in mitigation of Mn-induced α-synuclein accumulation and neuronal injury in the nigrostriatal regions of mice (Pajarillo et al., 2024). This suggests that enhancing REST might afford an effective strategy to develop therapeutics against Mn-induced neurotoxicity.

Tamoxifen (TX) is a selective estrogen receptor modulator (SERM), and its active metabolite, 4-hydroxytamoxifen, interacts with estrogen receptors (ER) to produce either agonistic or antagonistic effects, depending on the tissue type and cellular context (Maximov et al., 2013). For example, TX functions as an antagonist in breast tissue but exhibits agonistic effects in bone and uterine tissues (Gottardis et al., 1990; Vogel et al., 2006). Moreover, TX has shown protective effects in various neurological and neurodegenerative conditions, attenuating Mn-induced motor deficits in mice (Pajarillo et al., 2018), enhancing movement recovery in rat spinal cord injury (Colon and Miranda, 2016), promoting cognition in postmenopausal women (Newhouse et al., 2013), and improving memory in ovariectomized mice (Velazquez-Zamora et al., 2012). The protective effects of TX in the brain involve both ER-dependent and ER-independent mechanisms. TX reduces neuronal apoptosis and inflammation in ischemic rats via the ER-dependent pathway and its agonistic effects (Finney et al., 2021; Novick et al., 2020), while inducing antioxidative effects in spinal cord injury models through an ER-independent mechanism (Mosquera et al., 2014; Zhang et al., 2007). The mechanism by which TX induces protection is unclear, but studies have shown that TX can exert protection by attenuating oxidative damage in male rat striatum, which was extracellularly exposed to a PD neurotoxin, 1-methyl-4-phenylpyridinium (MPP+), via microdialysis (Obata and Kubota, 2001). Our previous studies showed that TX exerted protection against Mn-induced toxicity in ovariectomized female mice, partly through its antioxidant, anti-inflammatory, and anti-apoptotic effects (Pajarillo et al., 2018). Recently, TX has been shown to upregulate REST in CAD and SH-SY5Y cells, and it also mitigated Mn-induced neurotoxicity, parallel with attenuation of Mn-reduced REST (Digman et al., 2025). Given that TX upregulates REST and REST protects against Mn toxicity (Digman et al., 2025; Pajarillo et al., 2020b; Pajarillo et al., 2024), it is worth testing if REST contributes to TX’s neuroprotective mechanisms.

In addition to REST’s potential transcriptional involvement in Mn toxicity and TX’s protection mechanisms, epigenetic modifications such as histone acetylation may play a significant role in neuroprotection or neurotoxicity, including Mn-induced neurotoxicity (Ao et al., 2024; Guo et al., 2018; Zhang et al., 2017). Histone acetylation, regulated by HATs and HDACs, modulates gene expression at the epigenetic level. It is well established that impaired acetylation of lysine 27 on histone 3 (H3K27ac) is linked to neurodegeneration in AD and Mn-induced neurotoxicity in rats, which might regulate antioxidant gene expression (Ao et al., 2024; Marzi et al., 2018). However, whether TX’s neuroprotective effects against Mn toxicity involve histone acetylation, particularly H3K27ac, is unknown, prompting us to explore TX’s potential epigenetic mechanisms.

Since Mn toxicity preferentially damages dopaminergic neurons in the nigrostriatal regions, we investigated whether dopaminergic REST is critical for TX’s neuroprotective effects against Mn-induced neurotoxicity in these regions. To determine if dopaminergic REST is critical in TX’s protective effects, we deleted dopaminergic REST using dopamine transporter (DAT)-Cre-driven REST conditional knockout (cKO) mice. We used female mice in the present study, as we found TX-induced protection against Mn toxicity in WT female mice in our previous study (Pajarillo et al., 2018). Our findings reveal that TX afforded significant attenuation of Mn-induced neurotoxicity in REST cKO mice, but the TX-induced protective effects on several molecular targets, such as superoxide dismutase 2 (SOD2), catalase (CAT), and mitochondrial fusion protein OPA1, in WT were not apparent in dopaminergic REST-deleted female mice, suggesting that these genes require dopaminergic REST for TX-induced protection against Mn toxicity.

## 2 Materials and methods

### 2.1 Chemicals

Manganese chloride (MnCl_2_, Cat. # 244589) was obtained from MilliporeSigma (St. Louis, MO). Pellets of tamoxifen citrate (TX, Cat. # E-351) and its placebo (Cat. # C-111) were obtained from Innovative Research of America (Sarasota, FL). Antibodies for TH (sc-25269), Bax (sc-7480), CAT (sc-271803), OPA1 (sc-393296), CREB-binding protein (CBP)/p300 (sc-32244), ER-α (sc-542), ER-β (sc-6822), interleukin (IL)-1β (sc-52012), tumor necrosis factor (TNF)-α (sc-52746), glutamate transporter-1 (GLT-1, sc-365634), and β-actin (sc-47778) were obtained from Santa Cruz Biotechnology (Dallas, TX). Antibodies for acetylated H3K27 (H3K27ac, 4353T), acetylated H3K9/14 (H3K9/14ac, 9677S), and tri-methylated H3K36 (H3K36me3, 4909T) were purchased from Cell Signaling Technology (Danvers, MA). Antibodies for REST (07-579) used for immunoblotting, and ChIP-grade REST Ab (17-641) for the ChIP assay were obtained from MilliporeSigma. Antibodies for histone H3 (ab1791), glutamate/aspartate transporter 1 (GLAST, ab416), rabbit anti-mouse (ab6728), goat anti-rabbit (ab97051) conjugated with horseradish peroxidase, and goat anti-mouse conjugated with Alexa Fluor 568 (ab175473) were obtained from Abcam (Waltham, MA). The Fluoro-Jade^®^ C (FJC) ready-to-dilute staining kit (TR-100-FJ) was purchased from Biosensis (Thebarton, Australia).

### 2.2 Animals

All experimental procedures were approved by the Florida A&M University Institutional Animal Care and Use Committee (Protocol #023-05, Tallahassee, FL). We used dopaminergic neuron-specific REST KO mice and REST loxP (floxed) mice generated in our previous study (Pajarillo et al., 2024). Mice were kept in groups of five per cage, maintaining a 12-h light/dark cycle at a constant temperature of 22 °C ± 2 °C with *ad libitum* access to food and water. Eight-week-old female dopaminergic REST cKO, totaling 48 mice, and WT/REST loxP mice, totaling 30 mice, were used in the experiments. WT mice were used as a positive control for dopaminergic REST cKO mice. Sample sizes (n = 12 for REST cKO groups; n = 10 for WT groups) were determined by power analyses for behavioral outcomes (α = 0.05, power = 0.8–0.9), and tissue samples from three mice per group were used for molecular assays.

Dopaminergic REST cKO mice (n = 12/group) were separated randomly into four groups as follows: (1) placebo plus vehicle, (2) placebo plus Mn, (3) TX plus vehicle, and (4) TX plus Mn. WT mice (n = 10/group) were arranged into three groups as follows: (5) placebo, (6) placebo plus Mn, and (7) TX plus Mn. TX pellets (25 mg/pellet, 21 days release) and their placebo pellets were used and implanted as previously reported (Pajarillo et al., 2018). All pellets were implanted subcutaneously at the back of the neck of the anesthetized mice through a 0.5 cm incision. The pellets were inserted with tweezers into a small pocket formed by bluntly dissecting caudolaterally. The incision and the closed suture (made with wound clips) were performed using aseptic techniques to minimize the risk of infection.

Twenty-four hours after the implantation, mice received Mn daily (330 μg as Mn; 30 mg/kg as MnCl_2_; 1 μL per nostril in both sides) for 3 weeks (Pajarillo et al., 2020a). This dose increases brain Mn levels by up to 2-folds in C57BL/6 mice, closely mimicking the 3-fold increase observed in Mn-exposed nonhuman primates (Bowman and Aschner, 2014). Although REST regulates various genes (Sun et al., 2005; Bruce et al., 2009), there’s no evidence showing that REST modulates genes linked to Mn transport or accumulation. Moreover, even mutations of genes that affect and are risk factors for dopaminergic neurodegeneration, such as BTBD9, did not change Mn concentrations (Chen et al., 2022), rationalizing to use the same paradigm for Mn exposure in REST cKO mice. Distilled water served as the vehicle. To prevent Mn loss from the nostrils, mice were sedated with isoflurane for 3 min before and after administration.

### 2.3 Open-field, rotarod, and novel object recognition (NOR) tests

Behavioral tests were conducted 24 h after the last Mn exposure to evaluate the effects of Mn as previously described (Pajarillo et al., 2024). The open-field, rotarod, and NOR tests assessed locomotor activity, motor coordination, and cognition, respectively. The open-field test was conducted in a Plexiglas arena, and the movement of each mouse was tracked using Fusion SuperFlex software v6.25. Prior to testing, mice were acclimated to the arena for three consecutive days. The distance traveled, vertical activity, and stereotypy activity for each mouse were recorded for 30 min, and these parameters were compared between groups using statistical analysis.

The AccuRotor rotarod system was used to assess motor coordination (Pajarillo et al., 2024). Mice were trained for three consecutive days with three trials per session, and the latency to fall was recorded using Fusion Software v6.3. The measurements of motor coordination were taken for 650 s in mice that remained on the rod throughout the test, and the average duration for each group was used for comparisons.

For the NOR test (Pajarillo et al., 2022a), mice were acclimated to the open-field arena for three consecutive days before testing. During the trial, mice underwent a familiarization phase followed by the NOR test. In the familiarization phase, each mouse was exposed to two identical objects (familiar objects, FO) placed in the back corners of the arena for 10 min. After this, one FO was replaced with a novel object (NO), differing in texture, shape, and color. Mice were then placed in the center of the arena with one FO and one NO for 10 min, and the time spent exploring both objects was recorded using Fusion software. Mice remained in the chamber until they accumulated at least 30 s of object exploration (Clark et al., 2000). NO difference scores and discrimination ratios were calculated to compare groups. A positive NO difference score and discrimination ratio above 0.5 indicated normal memory and object retention, while negative scores and ratios below 0.5 suggested memory deficits (Bevins and Besheer, 2006).

### 2.4 Dissection and preparation of mouse brain tissue

After behavior tests, three animals per group were perfused transcardially with 4% (w/v) paraformaldehyde in 0.1 M phosphate-buffered saline (PBS) at pH 7.4 under anesthesia with ketamine/xylazine. Brains were removed and prepared for cryosectioning and immunohistochemistry. For cryosectioning, brains were post-fixed in a fixative overnight and transferred into a 30% w/v sucrose solution for cryoprotection. Serial coronal sections of 25-µm thickness were made with an HM525 NX Cryostat (Thermo Fisher Scientific, Waltham, MA). Coronal sections of substantia nigra (SN), spanning from Bregma −2.90 to −3.65 mm, were prepared for immunohistochemistry.

All processes for brain dissection were completed within a 2-h window (4:00 p.m.–6:00 p.m.). The time from decapitation to storing brain samples in dry ice was under 4 min. The striatum and midbrain were selected for study due to their relevance as primary sites of Mn accumulation and their critical roles in regulating movement and memory (Jay, 2003; Mizumori and Jo, 2013; Puig et al., 2014; Bradbury et al., 1985). After extracting the brain, these regions were dissected as described in previous protocols (Meyerhoff et al., 2021), then quickly frozen in dry ice and stored at −80 °C until further processing.

### 2.5 TH immunohistochemistry with FJC staining

TH expression was analyzed in coronal brain sections from placebo/TX plus vehicle/Mn-treated dopaminergic REST cKO mice. Frozen sections (25 µm) were washed 3 times with PBS for 5 min. After washing, sections were incubated with a blocking solution (PBS with 0.01% Triton X-100 and 2% normal goat serum) for 1 h at room temperature. The primary TH antibody was applied overnight at 4 °C at a 1:200 dilution. The sections were washed 3 times with PBS, followed by incubation with a goat anti-mouse IgG Alexa Fluor 568 secondary antibody in a blocking solution for 1 h at room temperature. After washing, the sections were processed for FJC staining of degenerated neurons according to the manufacturer’s instructions. The sections were then washed with PBS, followed by air-drying and mounting onto glass slides with mounting medium. High-resolution imaging was performed using a Nikon Eclipse Ts2R-FL microscope with a DS-Qi2 high-definition camera.

### 2.6 Real-time quantitative RT-PCR (qRT-PCR) analysis

Midbrain tissues (3 samples/group) were used to assess the mRNA levels of TH, REST, CAT, SOD1, SOD2, heme oxygenases 1 and 2 (HO-1 and HO-2), OPA1, peroxiredoxins 1 and 5 (PRDX1 and PRDX5), NADH:ubiquinone oxidoreductase subunit A2 (NDUFA2), IL-1β, TNF-α, GLAST, and GLT-1. Total RNA was extracted from the midbrain tissues using RNeasy^®^ Mini Kit (Qiagen, Valencia, CA), and reverse transcription was performed using the High-Capacity cDNA Reverse Transcription Kit (Applied Biosystems, Foster City, CA). For real-time qPCR, the following primers for mouse were used: TH, 5′-CAC TAT GCC CAC CCC CAG-3′ (forward) and 5′-CGC CGT CCA ATG AAC CTT-3′ (reverse); REST, 5′-ACT TTG TCC TTA CTC AAG CTC-3′ (forward) and 5′-CAT TTA AAT GGC TTC TCA CCT G-3′ (reverse); IL-1β, 5′-GAG GAC ATG AGC ACC TTC TTT-3′ (forward) and 5′-GCC TGT AGT GCA GTT GTC TAA-3′ (reverse); TNF-α, 5′-GAT GAG AAG TTC CCA AAT GGC-3′ (forward) and 5′-ACT TGG TGG TTT GCT ACG ACG-3′ (reverse); GLT-1, 5′-CTC ACT GAC TGT GTT TGG TG-3′ (forward) and 5′-GAG GTG CCA CCA GAA CTT TC-3′ (reverse); GLAST, 5′-GAT CGG AAA CAT GAA GGA GC-3′ (forward) and 5′-CAA GAA GAG GAT GCC CAG AG-3′ (reverse); CAT, 5′-ACC AGA TAC TCC AAG GCA AAG G-3′ (forward) and 5′-CCA GTG ACT GTG GAG AAT CGA A-3′ (reverse); SOD1, 5′-GGG GAC AAT ACA CAA GGC TGT A-3′ (forward) and 5′-GTC TCC AAC ATG CCT CTC TCT TCA-3′ (reverse); SOD2, 5′-CAG ACC TGC CTT ACG ACT ATG G-3′ (forward) and 5′-CTC GGT GGC GTT GAG ATT GTT-3′ (reverse); HO-1, 5′-AAG CGA GAA TGC TGA GTT CA-3′ (forward) and 5′-GCC GTG TAG ATA TGG TAC AAG GA-3′ (reverse); HO-2, 5′-TCG GAG GGG GTA GAT GAG TC-3′ (forward) and 5′-GCT TCC TTG GTC CCT TCC TT-3′ (reverse); OPA1, 5′-TGG AAA ATG GTT CGA GAG AGT CAG-3′ (forward) and 5′-CAT TCC GTC TCT AGG TTA AAG CG-3′ (reverse); PRDX1, 5′-AAT GCA AAA ATT GGG TAT CCT GC-3′ (forward) and 5′-CGT GGG ACA CAC AAA AGT AAA GT-3′ (reverse); PRDX5, 5′-TTC TGT GCT CCG TGC ATC G-3′ (forward) and 5′-TCC TGG TCC CCA GTT TCT GAT-3′ (reverse); NDUFA2, 5′-TTG CGT GAG ATT CGC GTT CA-3′ (forward) and 5′-ATT CGC GGA TCA GAA TGG GC-3′ (reverse); GAPDH, 5′-CTC ATG ACC ACA GTC CAT GC-3′ (forward) and 5′-CAC ATT GGG GGT AGG AAC AC-3′ (reverse). The total reaction volume (25 µL) contained 1 µL of cDNA template of each sample, 0.4 µM of primers, and iQ™ SYBR^®^ Green Supermix from Bio-Rad Laboratories, Inc. (Hercules, CA). The qPCR parameters were set for one cycle at 95 °C for 10 min, 40 cycles at 95 °C for 15 s, and 60 °C for 1 min in the Bio-Rad CFX96 real-time PCR system. GAPDH was used to normalize all samples. Data analysis was performed using the Bio-Rad CFX Manager version 3.1.

### 2.7 Immunoblotting

The harvested tissues from the striatum and midbrain were homogenized and used for protein extraction and further analysis. A 3:1 ratio of RIPA buffer with protease inhibitors to each tissue was used, and the protein concentration was determined by bicinchoninic acid assay. Ten µg of total protein per lane was run on 10% SDS-PAGE gels and transferred to a nitrocellulose membrane for immunoblotting with relevant antibodies. All blots were developed using the SuperSignal™ West Pico PLUS chemiluminescent substrate kit, followed by blot imaging and quantification with the Molecular Imager ChemiDoc™ XRS+ System (Bio-Rad).

### 2.8 ChIP assay

Following the manufacturer’s protocol, the ChIP assay was conducted using the EZ-ChIP kit (MilliporeSigma). Briefly, midbrain tissues were crosslinked with formaldehyde for 10 min at room temperature and then washed with ice-cold PBS. Tissues were lysed in SDS lysis buffer with protease inhibitors, sonicated, and centrifuged at 15,000 × g for 10 min at 4 °C. The supernatant was diluted and incubated with protein G agarose beads for 1 h at 4 °C. After incubation, 1% of the supernatant was saved as input, and the remainder was incubated overnight with H3K27ac, REST, and rabbit IgG (negative control) antibodies at 4 °C. Protein G agarose beads were added and incubated for 1 h, followed by washing with low and high salt buffers. Following reverse crosslinking, immunoprecipitated DNA was purified and analyzed by qPCR using promoter-specific primers for mouse: TH, 5′-CCC ATA TGC CCT GGT TTG AT-3′ (forward) and 5′-AGG CCT CCG TCC CAT TAG AT-3′ (reverse); REST, 5′-CCA ACT TTT CCC CGC TCT-3′ (forward) and 5′-GCG TCC TAC CCT CTG AAC TG-3′ (reverse); CAT, 5′-AAA TAA GCT GCA AAG CCA CCA A-3′ (forward) and 5′-CAT AGC TCC TTT GAG ACC AGA C-3′ (reverse); SOD2, 5′-GAT GAA CAC ACG CAA ACC TG-3′ (forward) and 5′-CTG GGA AAC CCT GGA GAC TT-3′ (reverse); OPA1, 5′-CTT GTT GCT GAG GGC CTC TT-3′ (forward) and 5′-CCA GAG CAG ACC ACA CAC AG-3′ (reverse). DNA was quantified using Bio-Rad CFX Manager 3.1 software, and results were expressed as enrichment of H3K27ac or REST binding at the selected gene promoters.

### 2.9 Statistical analysis

All data were expressed as the mean ± standard deviation (SD). Multiple comparison analyses were performed by a two-way analysis of variance (ANOVA) followed by Fisher LSD *post hoc* tests using the GraphPad Prism Software version 10.0 (San Diego, CA). A *p-value* of <0.05 was considered statistically significant.

## 3 Results

### 3.1 TX attenuated Mn-induced impairment of movement and motor coordination in dopaminergic REST-deleted (REST cKO) mice

Since TX exerted neuroprotection against Mn toxicity in ovariectomized WT female mice (Pajarillo et al., 2018) and dopaminergic REST is critical for neuroprotection against Mn-induced toxicity in mice (Pajarillo et al., 2024), first, we tested whether dopaminergic REST is critical for the neuroprotective effects of TX against Mn toxicity.

REST cKO and WT female mice were used for molecular biology experiments. Mice were not ovariectomized in the present study, as they undergo estrous cycles every 4 to 5 days during the 21-day experimental period. This Mn exposure paradigm is different from our previous studies in which a single dose of Mn was given to the ovariectomized mice (Pajarillo et al., 2018). REST cKO mice were divided into four groups (two vehicle groups and two Mn-exposed groups), with one group in each receiving a TX pellet implant 1 day prior to Mn exposure. Mn was administered daily via intranasal instillation (330 μg as Mn, 30 mg/kg as MnCl_2_) for 3 weeks, while control mice received distilled water.

**FIGURE 1:**
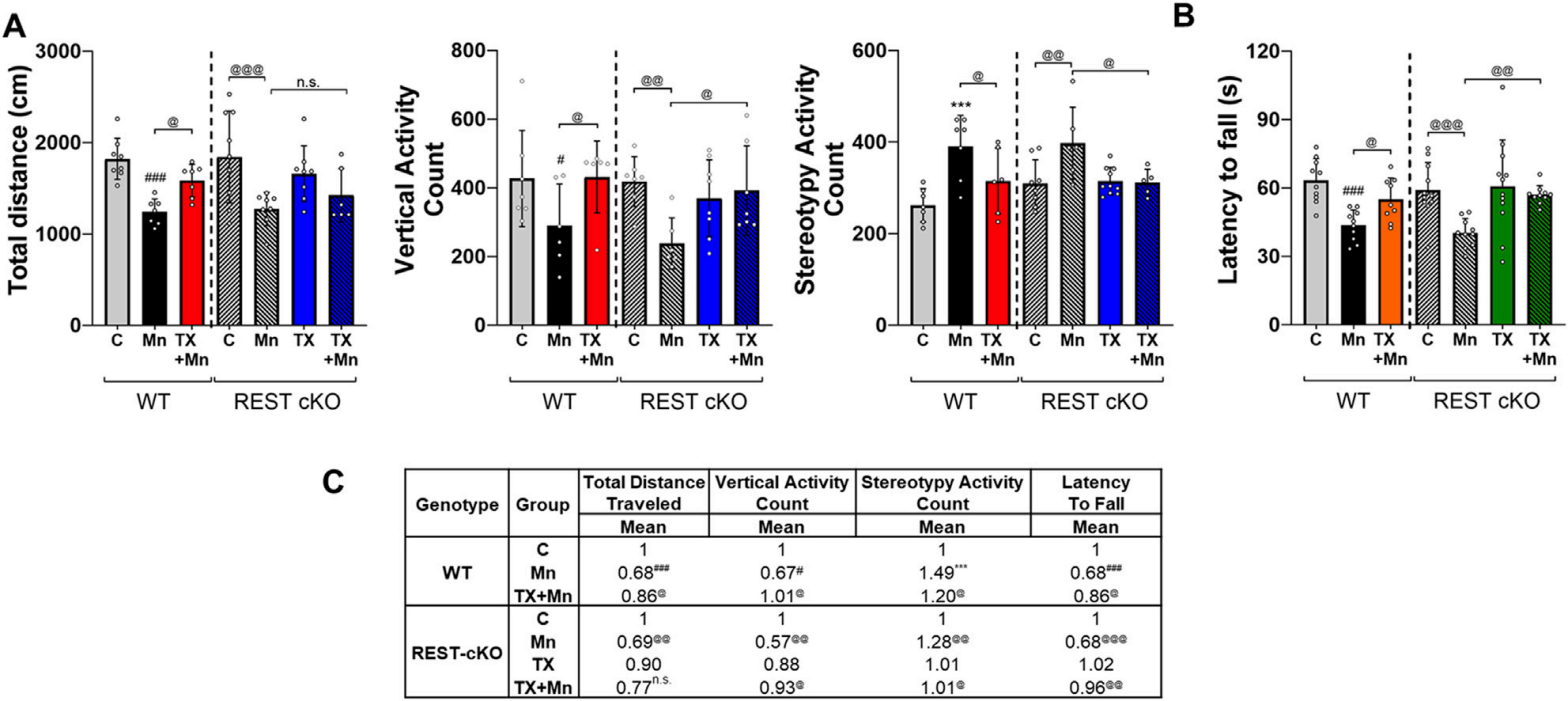
TX attenuated Mn-induced impairment of locomotor activity in female WT and REST cKO mice. **(A)** Following TX pellet implantation and Mn exposure as described in the Methods, locomotor activity was measured, and various locomotor activity parameters were quantified. Total distance traveled, vertical activity, and stereotypy activity were assessed. **(B)** Motor coordination was measured by fall latency; the time spent on the rotating rod. **(C)** The mean fold change of locomotor activity parameters has been calculated for each group. *n.s.*, not significant; ***p < 0.001, ^#^ *p* < 0.05, ^###^ *p* < 0.001, compared with the controls; ^@^ *p* < 0.05, ^@@^ *p* < 0.01, ^@@@^ *p* < 0.001, compared with each other (two-way ANOVA followed by Fisher LSD; n = 5). Data are expressed as mean ± SD.

To assess TX effects on behavioral changes in Mn-exposed mice, 24 h after the last Mn exposure, locomotor activities and motor coordination were assessed by open-field and rotarod tests, respectively, in WT and REST cKO mice. Results revealed that Mn decreased locomotor activities in the open field arena, while TX attenuated several parameters of Mn-induced motor deficits in WT and REST cKO mice (Figure 1). Mn decreased total distance traveled, vertical activity, and stereotypy activity (Figure 1A). Importantly, TX did not attenuate the Mn-induced decrease in total distance traveled in REST cKO mice, unlike its protective effects in WT mice (Figure 1A). However, TX attenuated Mn-decreased vertical activity and Mn-increased stereotypy activity in REST cKO mice, as well as in WT mice (Figures 1A,C). In addition, Mn decreased the latency to fall, corroborating impairment of motor coordination, while TX mitigated this Mn-induced effect in both WT and REST cKO mice (Figures 1B,C).

### 3.2 TX attenuated Mn-impaired cognitive function in REST CKO mice

**FIGURE 2:**
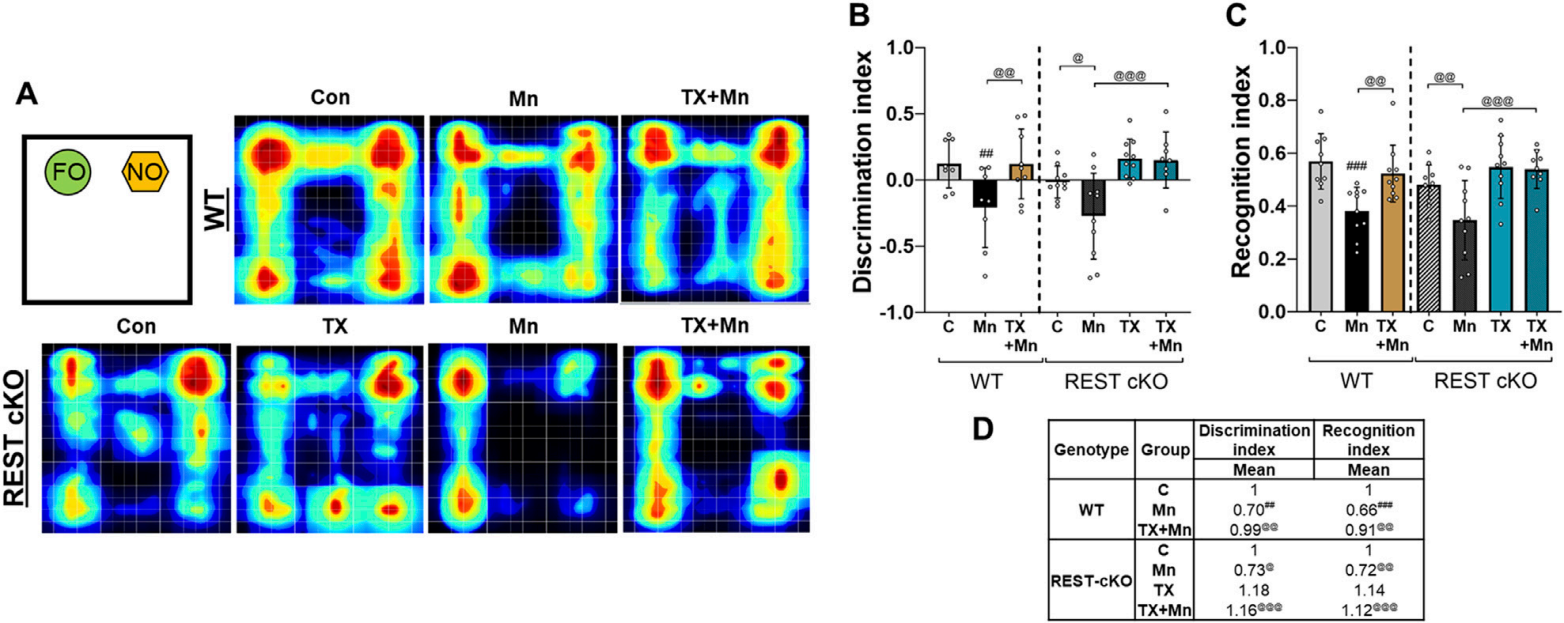
TX attenuated Mn-impaired cognition in WT and REST cKO mice. Following TX pellet implantation and Mn exposure as described in the Methods, the NOR test was conducted. Mice were allowed to explore a FO (top left) and a NO (top right), and their exploration behavior was recorded. **(A)** Heatmaps show the time each mouse spent in different areas of the arena during the 10-min session (blue to red indicates increasing time). **(B**,**C)** The time spent interacting with each object was used to calculate discrimination **(B)** and NOR **(C)** indices. **(D)** The mean fold change of NOR test parameters has been calculated for each group. ^##^ *p* < 0.01, ^###^ *p* < 0.001, compared with the controls; ^@^ *p* < 0.05, ^@@^ *p* < 0.01, ^@@@^ *p* < 0.001, compared with each other (two-way ANOVA followed by Fisher LSD; n = 5). Data are expressed as mean ± SD.

The NOR test was performed to assess the effects of TX on Mn-induced cognitive impairment in REST cKO mice. Given that TX attenuated Mn-induced memory and learning deficits in rats (Owoyemi et al., 2024), we tested if dopaminergic REST plays a role in TX’s attenuation of Mn-induced cognitive impairment in mice. Results revealed that Mn significantly decreased the recognition and discrimination indices, corroborating impaired cognitive function, while TX treatment mitigated these Mn-induced cognitive deficits in WT and REST cKO mice (Figures 2A–D), indicating that TX attenuated Mn-induced impairment in cognition in the absence of dopaminergic REST.

### 3.3 TX mitigated Mn-induced dopaminergic neurotoxicity and REST dysregulation in the nigrostriatal region of REST cKO mice

**FIGURE 3:**
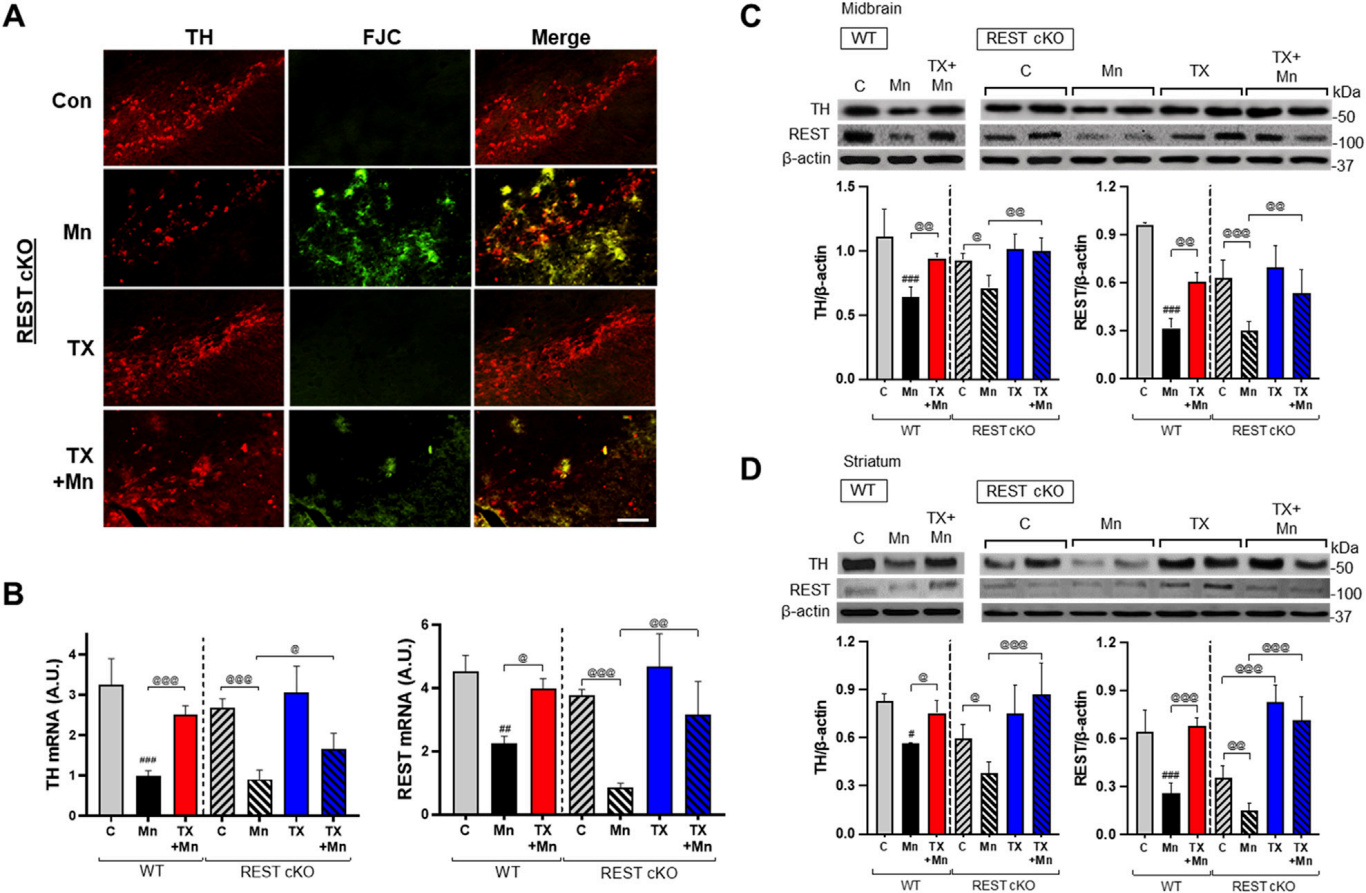
TX attenuated Mn-induced dopaminergic toxicity in the nigrostriatal regions of REST cKO mice. After TX pellet implantation and Mn exposure as described in the Methods, **(A)** coronal sections of the SN were co-stained with FJC and TH, a marker of dopaminergic neurons, by IHC (scale bar = 200 µm). **(B**,**C)** The mRNA and protein levels of TH and REST were measured in the midbrain. **(D)** Protein levels of TH and REST were analyzed in the striatum. GAPDH and β-actin were used as loading controls for mRNA and protein, respectively. ^#^ *p* < 0.05, ^##^ *p* < 0.01, ^###^ *p* < 0.001, compared with the controls; ^@^ *p* < 0.05, ^@@^ *p* < 0.01, ^@@@^ *p* < 0.001, compared with each other (two-way ANOVA followed by Fisher LSD; n = 3). Data are expressed as mean ± SD.

Mn caused neuronal damage as evidenced by increased FJC staining and decreased TH protein levels in the SN where dopaminergic cell bodies are located in the nigrostriatal pathway of REST cKO mice (Figure 3A). This Mn-induced decrease in TH immunofluorescence in the SN paralleled the decrease in TH and REST mRNA and protein levels in the midbrain and striatum of REST cKO mice (Figures 3B–D). TX alone increased REST protein levels in the striatum of REST cKO (Figure 3D), indicating that TX exerts an agonistic effect to increase REST, possibly in non-dopaminergic and other neural cell types.

### 3.4 TX mitigated Mn-induced dysregulation of proapoptotic Bax in REST cKO mice

**FIGURE 4:**
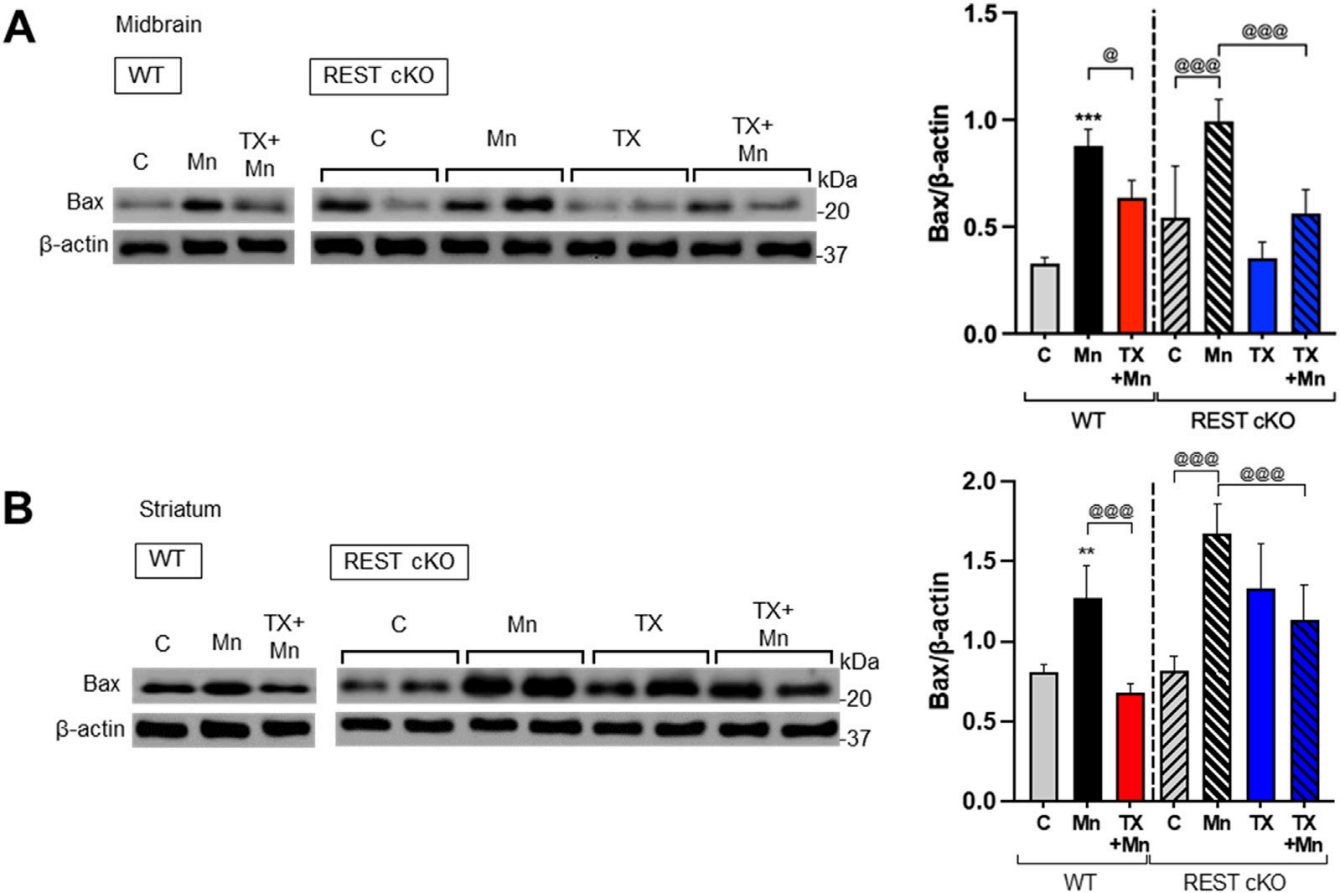
TX attenuated Mn-increased proapoptotic Bax levels in the nigrostriatal regions of REST cKO mice. After TX pellet implantation and Mn exposure as described in the Methods, midbrain and striatum samples were processed for immunoblotting. **(A**,**B)** Proapoptotic Bax protein levels in the midbrain **(A)** and striatum **(B)** were determined. β-actin was used as a loading control for protein. **p < 0.01, ***p < 0.001, compared with the controls; ^@^ *p* < 0.05, ^@@@^ *p* < 0.001, compared with each other (two-way ANOVA followed by Fisher LSD; n = 3). Data are expressed as mean ± SD.

To further determine if TX induces neuroprotection against Mn toxicity in the dopaminergic REST-deleted mice, we examined TX’s modulatory effects on Mn-induced apoptosis by measuring the protein levels of proapoptotic Bax in both WT and REST cKO mice. The results revealed that Mn increased Bax levels in both midbrain and striatum in WT as well as REST cKO mice, with a further increase in cKO, while TX significantly attenuated this Mn-induced effect in both WT and REST cKO (Figures 4A,B).

### 3.5 Dopaminergic REST played a role in TX’s attenuation of Mn-decreased antioxidant genes in mice

**FIGURE 5:**
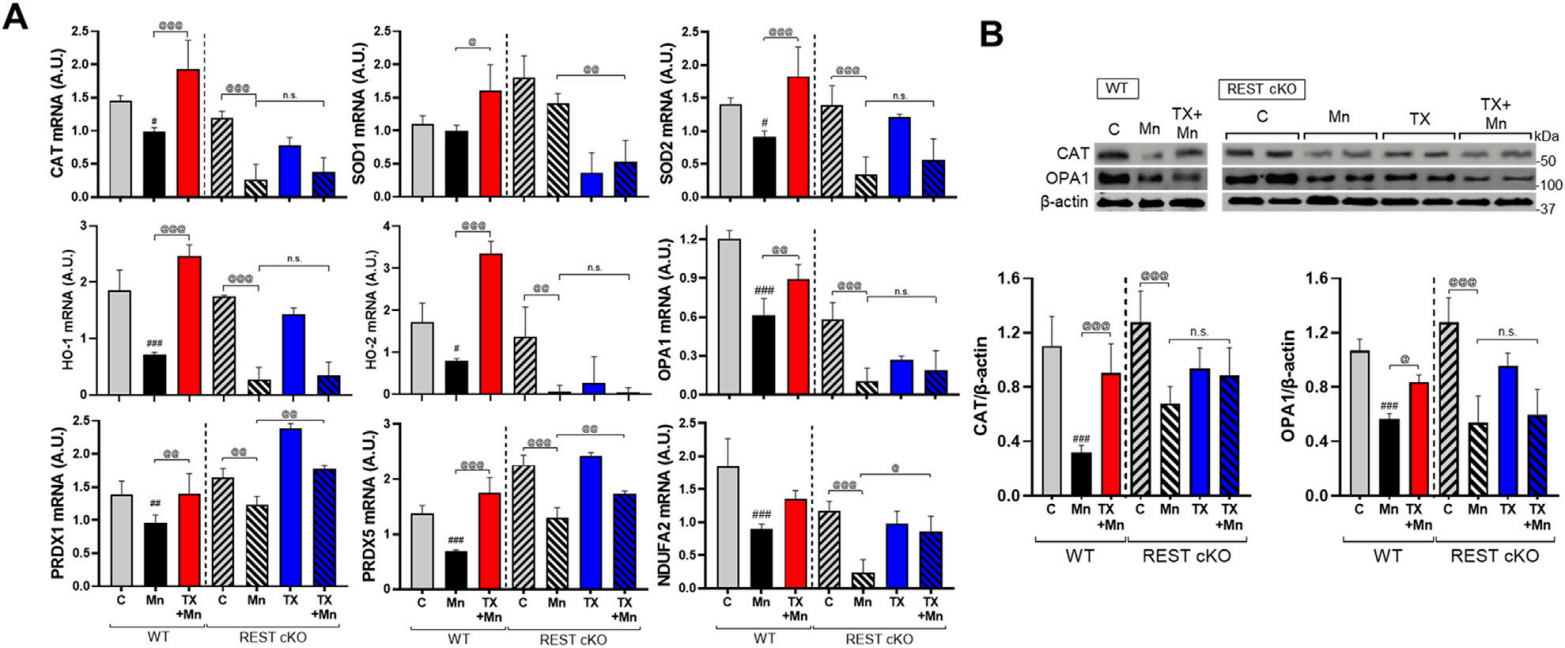
TX did not attenuate several Mn-decreased antioxidant and mitochondrial genes in the midbrain of REST cKO mice. After TX pellet implantation and Mn exposure as described in the Methods, midbrain tissues were processed for qPCR and immunoblotting. **(A)** The mRNA levels of CAT, SOD1/2, HO-1/2, OPA1, PRDX1/5, and NDUFA2 were compared between groups. **(B)** Protein levels of CAT and OPA1 were analyzed in the midbrain. GAPDH and β-actin were used as loading controls for mRNA and protein, respectively. *n.s.*, not significant; ^#^ *p* < 0.05, ^##^ *p* < 0.01, ^###^ *p* < 0.001, compared with the controls; ^@^ *p* < 0.05, ^@@^ *p* < 0.01, ^@@@^ *p* < 0.001, compared with each other (two-way ANOVA followed by Fisher LSD; n = 3). Data are expressed as mean ± SD.

Since Mn caused oxidative stress in dopaminergic neuronal cultures and the nigrostriatal regions of mice, and TX, as well as REST, attenuated these Mn-induced effects (Pajarillo et al., 2018; Pajarillo et al., 2024; Pajarillo et al., 2020b), we investigated whether dopaminergic REST plays a role in TX’s protective effects against Mn-induced oxidative stress. Our analyses focused on the substantia nigra pars compacta in the midbrain region, where dopaminergic cell bodies are localized and dopaminergic REST was selectively deleted. This REST deletion will likely exert greater transcriptional and epigenetic effects than the striatum, which may be less affected. Several antioxidant genes, including CAT, SOD1/2, HO-1/2, and PRDX1/5, were assessed. In addition, since Mn causes mitochondrial damage (Liccione and Maines, 1988), we assessed the mitochondrial protein NDUFA2, a subunit of NADH dehydrogenase located in the mitochondrial inner membrane, and mitochondrial fusion protein OPA1. The results demonstrated that Mn exposure led to reduced mRNA expression of antioxidant genes (CAT, SOD2, HO-1/2, and PRDX1/5) and mitochondrial genes (OPA1 and NDUFA2) in the midbrain of both WT and REST cKO mice, with more pronounced decreases observed in REST cKO mice for most of the genes examined (Figure 5A). Importantly, TX’s attenuation of Mn-dysregulated antioxidant and mitochondria-related genes was differentially regulated in WT and REST cKO mice. TX attenuated Mn-induced dysregulation of all genes tested above in WT mice (Figures 5A–C). In REST cKO mice, on the other hand, TX significantly restored the mRNA levels of antioxidant PRDX1, PRDX5, and mitochondrial NDUFA2, but failed to attenuate the Mn-induced decreases in antioxidants CAT, SOD1/2, HO-1/2, and mitochondrial OPA1 (Figure 5A). TX was also unable to attenuate Mn-decreased protein levels of CAT and OPA1 in the midbrain of REST cKO mice, similar to its effects on mRNA levels of those (Figure 5B).

### 3.6 TX mitigated the Mn-induced increase in proinflammatory cytokines IL-1β and TNF-α in REST cKO mice

**FIGURE 6:**
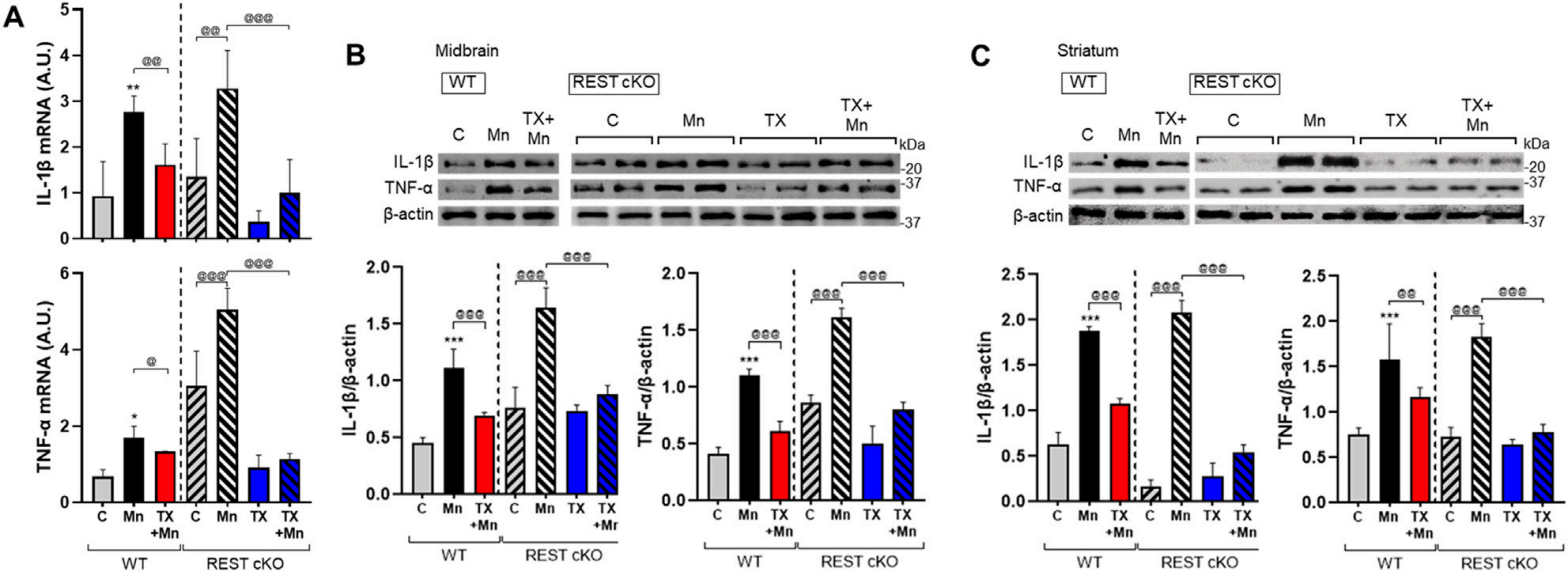
TX attenuated the Mn-induced increase of proinflammatory cytokines in the nigrostriatal regions of REST cKO mice. After TX pellet implantation and Mn exposure as described in the Methods, tissue samples were processed for qPCR and immunoblotting. **(A)** The mRNA levels of IL-1β and TNF-α were analyzed in the midbrain. **(B**,**C)** Protein levels of IL-1β and TNF-α were assessed in the midbrain **(B)** and striatum **(C)**. GAPDH and β-actin were used as loading controls for mRNA and protein, respectively. *p < 0.05, **p < 0.01, ***p < 0.001, compared with the controls; ^@^ *p* < 0.05, ^@@^ *p* < 0.01, ^@@@^ *p* < 0.001, compared with each other (two-way ANOVA followed by Fisher LSD; n = 3). Data are expressed as mean ± SD.

Since inflammation plays a critical role in neurotoxicity, we tested if TX attenuates Mn-induced inflammation and the role of dopaminergic REST in these TX effects. The expression levels of proinflammatory cytokines IL-1β and TNF-α in the nigrostriatal regions of WT and REST cKO mice were assessed. These brain regions contain other types of neural cells, not just dopaminergic neurons, as well as non-dopaminergic neurons, and glial cells. Thus, inflammatory effects could be derived from all neural cell types. Results showed that Mn increased mRNA and protein levels of IL-1β and TNF-α in both the midbrain and striatum of WT and REST cKO mice, with further increases in REST cKO mice (Figures 6A–C). However, TX attenuated these Mn-induced effects regardless of genotype, indicating that dopaminergic REST did not play a critical role in TX’s anti-inflammatory effects against Mn.

### 3.7 TX mitigated the Mn-induced decrease in astrocytic glutamate transporters in REST cKO mice

**FIGURE 7:**
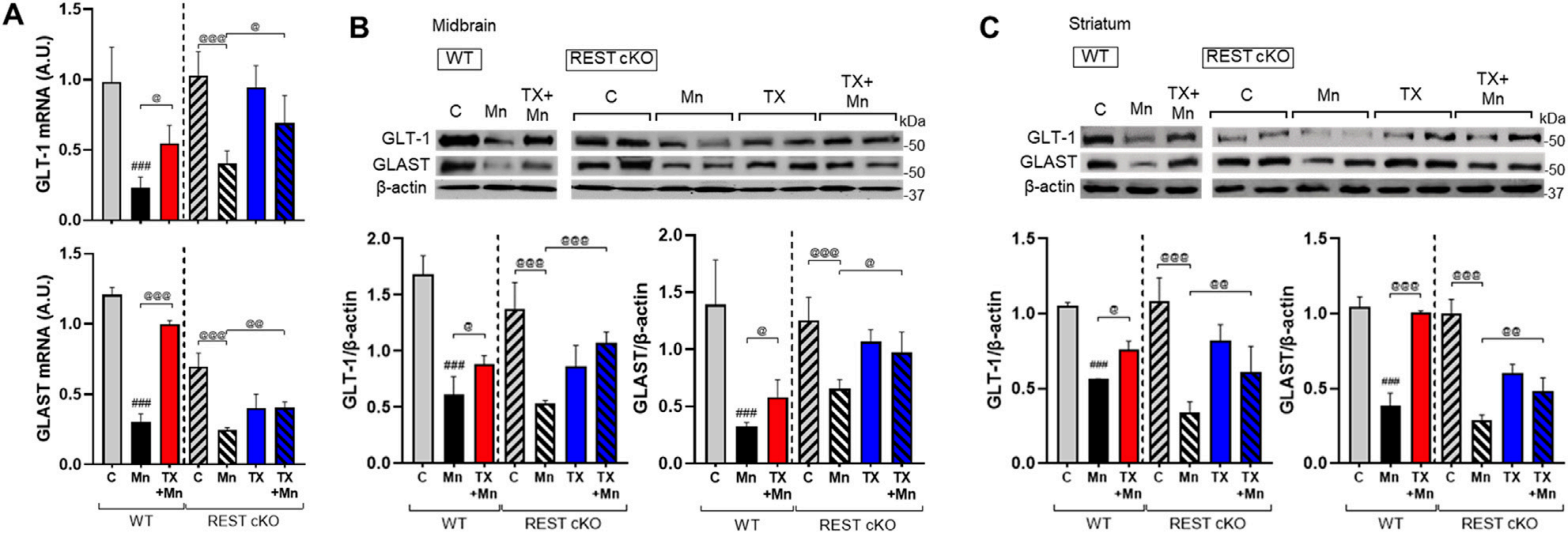
TX attenuated Mn-reduced astrocytic glutamate transporters in the midbrain of REST cKO mice. After TX pellet implantation and Mn exposure as described in the Methods, tissue samples were processed for qPCR and immunoblotting. **(A)** The mRNA levels of GLT-1 and GLAST were analyzed in the midbrain. **(B**,**C)** Protein levels of GLT-1 and GLAST were assessed in the midbrain **(B)** and striatum **(C)**. GAPDH and β-actin were used as loading controls for mRNA and protein, respectively. ^###^ *p* < 0.001, compared with the controls; ^@^ *p* < 0.05, ^@@^ *p* < 0.01, ^@@@^ *p* < 0.001, compared with each other (two-way ANOVA followed by Fisher LSD; n = 3). Data are expressed as mean ± SD.

Mn-impaired astrocytic glutamate transporters, GLAST and GLT-1, which TX attenuated in rat astrocyte cultures (Lee et al., 2009) as well as ovariectomized mice (Pajarillo et al., 2018). Thus, we further tested if dopaminergic REST plays a role in TX’s attenuation against Mn-induced dysregulation of GLT-1 and GLAST in the nigrostriatal region of WT and REST cKO mice, possibly via indirect mechanisms such as elevated glutamate levels (Kim et al., 2025) and oxidative stress. Results showed that TX was able to attenuate the Mn-induced decrease in mRNA and protein levels of the GLAST and GLT-1 similarly in both WT and REST cKO mice (Figures 7A–C), indicating that dopaminergic REST may not contribute to TX’s protective effects against Mn-induced reduction in GLT-1 and GLAST.

### 3.8 Dopaminergic REST played a role in TX effects on Mn-induced epigenetic dysregulation in mice

**FIGURE 8:**
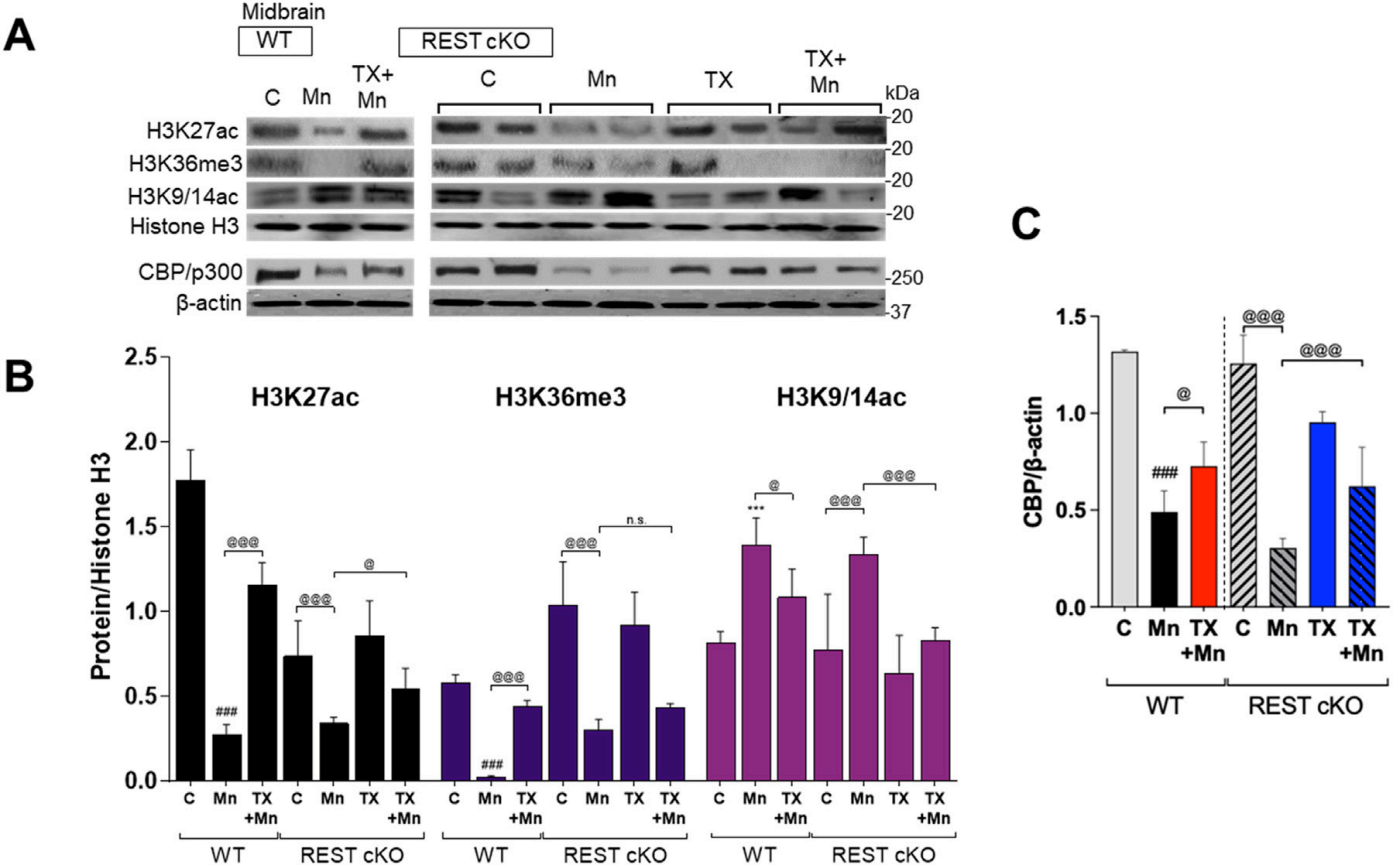
TX modulated Mn-dysregulated acetylated histone marks for gene activation and CBP/p300 in the midbrain of REST cKO mice. After TX pellet implantation and Mn exposure as described in the Methods, midbrain samples were processed for immunoblotting. **(A–C)** Levels of acetylation of histone H3 lysine 27 (H3K27ac), lysines 9 and 14 (H3K9/14ac), and tri-methylation levels of histone H3 lysine 36 (H3K36me3) were determined in the midbrain of REST cKO mice. **(C)** CBP/p300 protein levels were measured in the midbrain. Histone H3 or β-actin was used as a loading control. *n.s.*, not significant; ***p < 0.001, ^###^ *p* < 0.001, compared with the controls; ^@^ *p* < 0.05, ^@@@^ *p* < 0.001, compared with each other (two-way ANOVA followed by Fisher LSD; n = 3). Data are expressed as mean ± SD.

Since TX attenuated Mn-dysregulated expression of several genes involved in neuroprotection, including TH, REST, and antioxidant genes at the transcriptional levels, we further investigated whether epigenetic alteration via histone modification contributed to those transcriptional changes induced by Mn and/or TX and potentially by dopaminergic REST deletion. Midbrain tissues were used since transcriptional regulations mostly occur in this region, where the cell bodies of dopaminergic neurons are localized. We assessed acetylation of histone marks, such as histones H3 lysine 27 (H3K27ac), lysine 9 and 14 (H3K9/14ac), and tri-methylation of histone H3 lysine 36 (H3K36me3) in the midbrain of WT and REST cKO mice. Results showed that the basal levels of H3K27ac are lower in REST cKO mice compared to WT mice, but Mn decreased H3K27ac in the midbrain of both WT and REST cKO mice (Figures 8A,B), which TX significantly attenuated in both genotypes. On the other hand, the basal levels of H3K36me3 were higher in REST cKO compared to WT, and Mn markedly decreased H3K36me3 levels, which were significantly attenuated by TX in WT, but not in REST cKO mice. Mn increased H3K9/14ac in the midbrain of both WT and REST cKO mice, which TX attenuated (Figures 8A,B).

We further tested if the histone-acetylating enzyme CREB-binding protein (CBP)/p300 plays a role in Mn and TX’s modulation of histone acetylation by measuring the protein levels of CBP/p300 in the midbrain of WT and REST cKO mice. Results showed that Mn markedly decreased CBP/p300 protein levels, which TX attenuated in both WT and REST cKO mice (Figures 8A,C).

**FIGURE 9:**
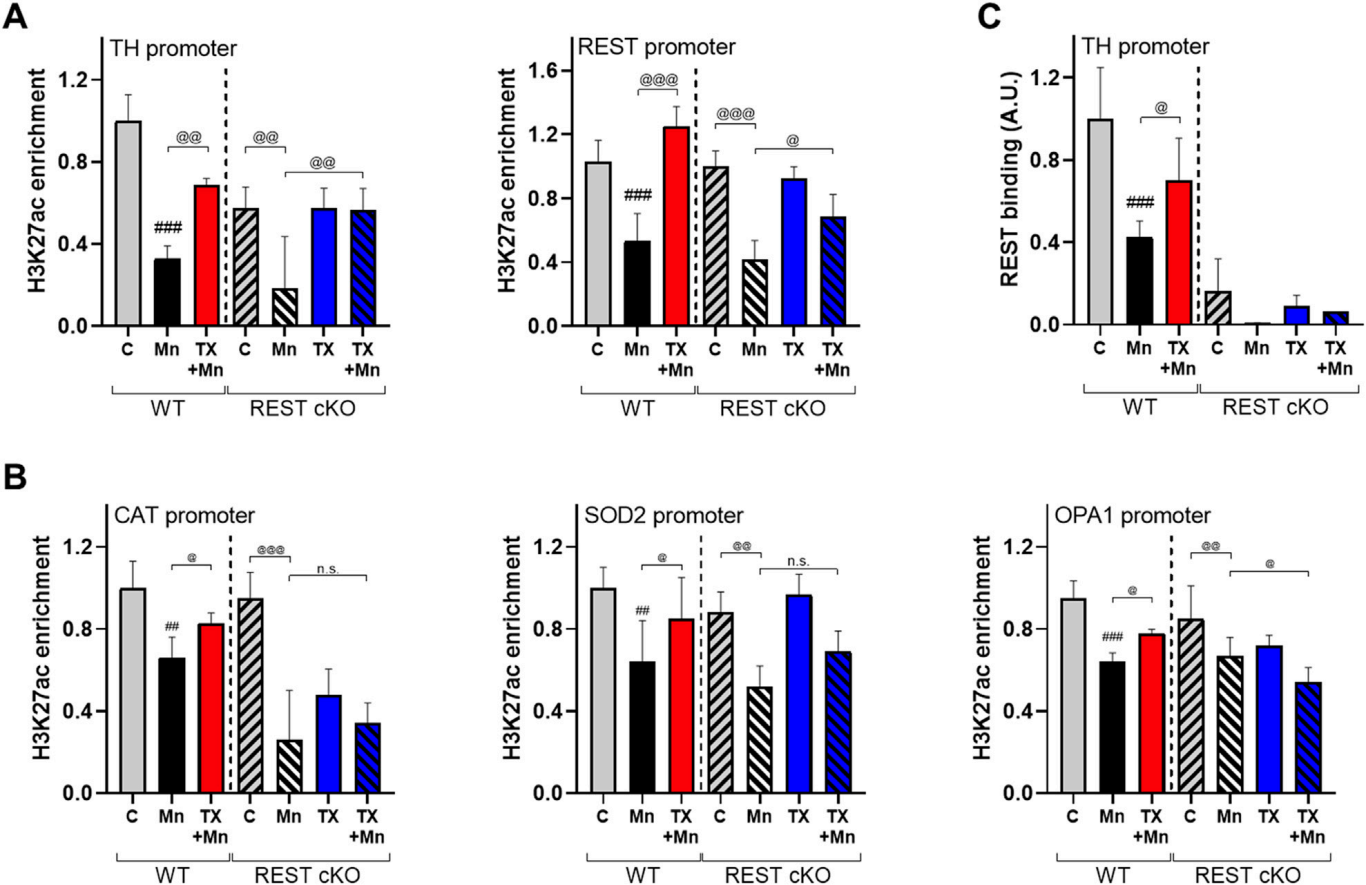
TX modulated Mn-dysregulated H3K27ac interaction with the promoters of TH, REST, CAT, SOD2, and OPA1 in the midbrain of REST cKO mice. After TX pellet implantation and Mn exposure, midbrain samples were processed for ChIP assay as described in the Methods. **(A**,**B)** ChIP assays followed by qPCR to measure the **(A)** enrichment levels of H3K27ac in the TH and REST promoters, and **(B)** enrichment levels of H3K27ac in the promoters of antioxidant genes CAT and SOD2 and the mitochondrial fusion protein OPA1. **(C)** The binding of REST to the TH promoter. ^##^ *p* < 0.01, ^###^ *p* < 0.001, compared with the controls; ^@^ *p* < 0.05, ^@@^ *p* < 0.01, ^@@@^ *p* < 0.001, compared with each other (two-way ANOVA followed by Fisher LSD; n = 3). Data are expressed as mean ± SD.

TX’s attenuation of Mn-decreased H3K27ac, which is known to upregulate multiple genes, including antioxidant genes (Yang et al., 2022; Ao et al., 2024), could mitigate Mn-induced transcriptional repression of the antioxidant genes tested. Accordingly, we determine if H3K27ac contributes to Mn/TX-regulation of TH, REST, and genes involved in oxidative stress and mitochondrial dysfunction. We assessed the interaction of H3K27ac with the promoters of TH, REST, CAT, SOD2, and OPA1 by ChIP assays in the midbrain of WT and REST cKO mice. Results showed that TX attenuated the Mn-decreased interaction of H3K27ac with the TH as well as REST promoters in both WT and REST cKO mice (Figure 9A). TX attenuated Mn-induced decrease in interaction of H3K27ac with the REST promoter in the midbrain of REST cKO mice (Figure 9A), possibly due to the presence of the REST promoter in other neural cell types and/or presence of dopaminergic REST gene sequences may still be present in the REST cKO, which has only REST’s exon 4 deletion (Gao et al., 2011).

Importantly, TX attenuated Mn-reduced interaction of H3K27ac with the promoters of CAT, SOD2, and OPA1 in WT, not REST cKO mice (Figure 9B), indicating that dopaminergic REST plays a critical role in the TX/Mn-induced modulation of H3K27ac’s interaction with these antioxidant genes. Since REST protein is deleted in dopaminergic neurons in REST cKO mice, REST binding to the TH promoter was almost completely absent (Figure 9C).

### 3.9 TX attenuated Mn-decreased ER-α and ER-β in REST cKO mice

**FIGURE 10:**
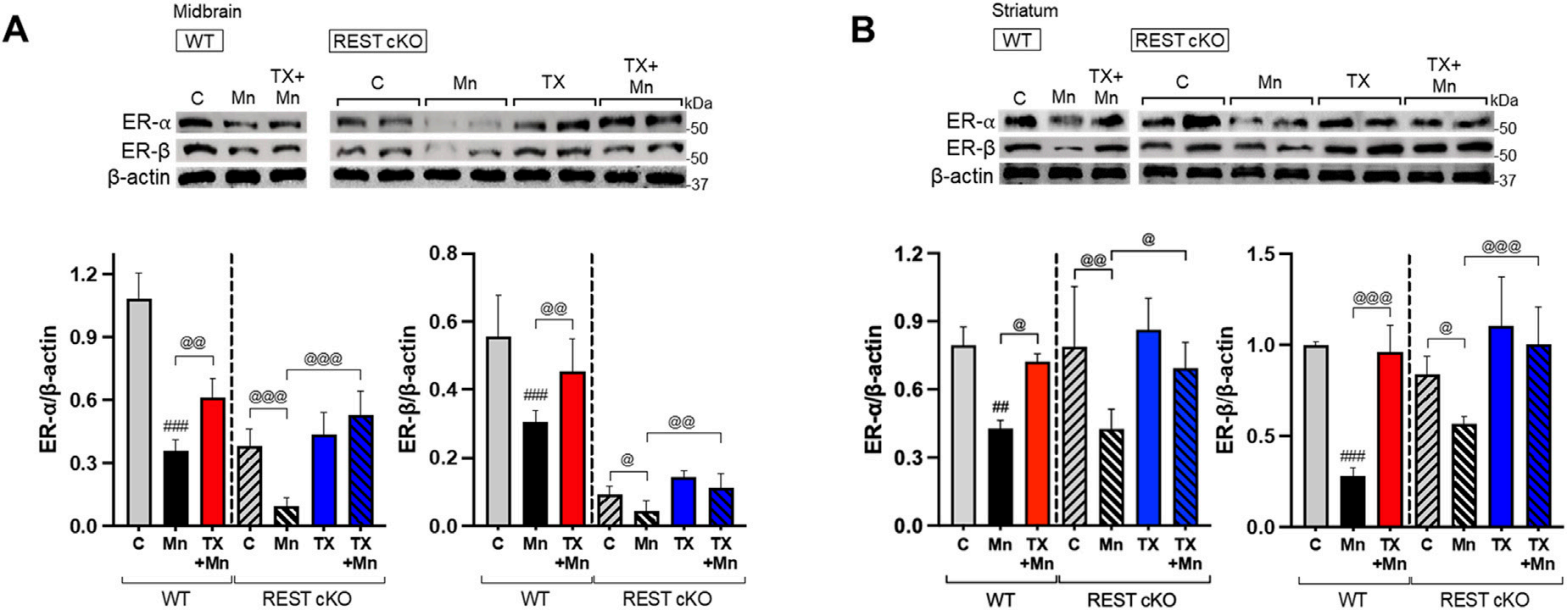
TX attenuated Mn-decreased levels of estrogen receptors in the midbrain of REST cKO mice. After TX pellet implantation and Mn exposure, tissue samples were processed for qPCR and immunoblotting as described in the Methods. **(A**,**B)** Protein levels of ER-α and ER-β in the midbrain **(A)** and the striatum **(B)** were compared across groups by immunoblotting. β-actin was used as a loading control. ^##^ *p* < 0.01, ^###^ *p* < 0.001, compared with the controls; ^@^ *p* < 0.05, ^@@^ *p* < 0.01, ^@@@^ *p* < 0.001, compared with each other (two-way ANOVA followed by Fisher LSD; n = 3). Data are expressed as mean ± SD.

Our previous studies have shown that Mn decreased ER-α protein levels, which TX attenuated in WT female mice (Pajarillo et al., 2018). In the present study, we determined if dopaminergic REST played a role in these TX or Mn effects on the ERs. Thus, ER-α and ER-β protein levels were analyzed in the nigrostriatal region of both WT and REST cKO mice. Mn significantly decreased ER-α and ER-β protein levels in these regions of both WT and REST cKO mice, while TX attenuated these Mn-induced reductions, indicating that dopaminergic REST was not involved in TX’s attenuation of Mn-reduced ER-α and ER-β protein levels (Figures 10A,B).

## 4 Discussion

**FIGURE 11:**
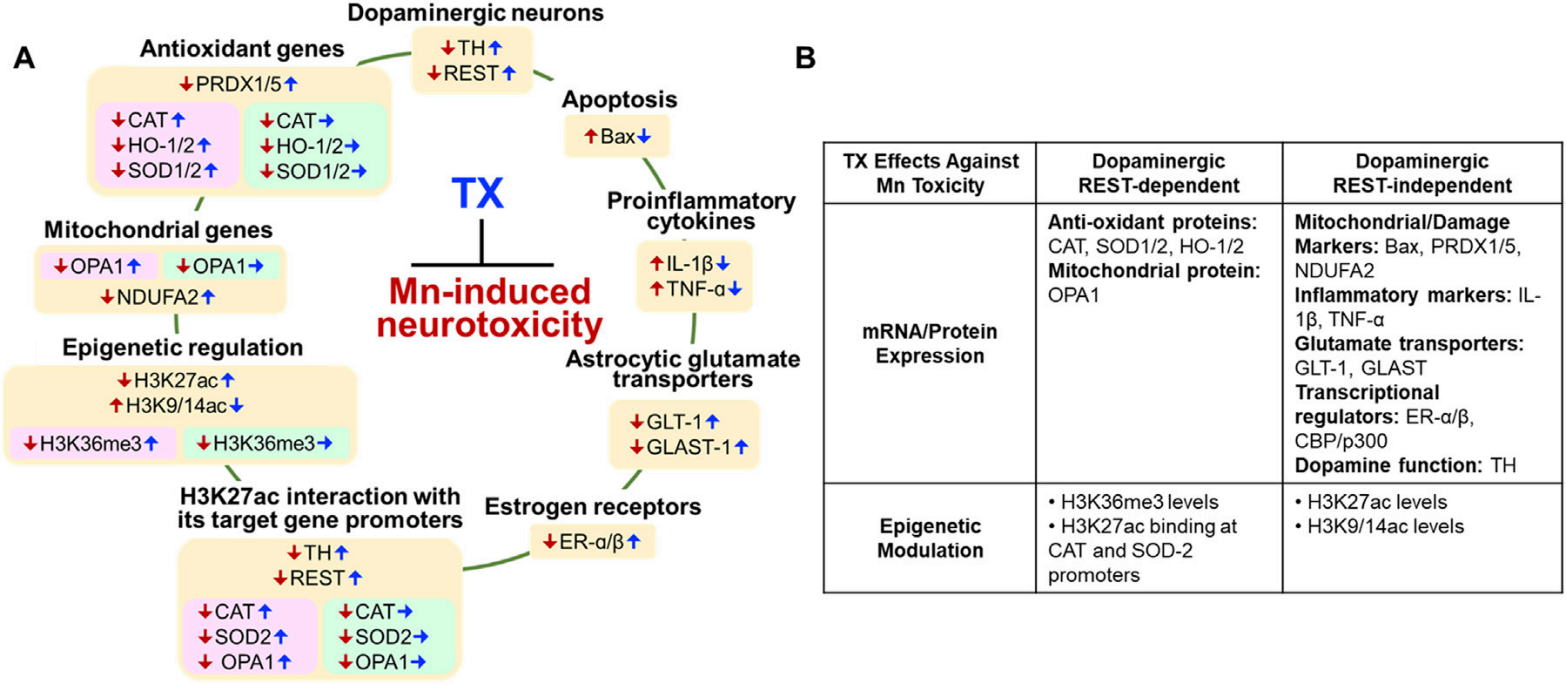
Proposed mechanisms of dopaminergic REST involvement in the TX’s protection against Mn-induced neurotoxicity and behavioral deficits in female mice. **(A)** Color-coded summary of TX effects across genotypes. Light peach yellow boxes represent TX’s protective effects in both WT and dopaminergic REST cKO mice. Light pink boxes indicate TX’s protective effects observed only in WT mice, while light green boxes indicate that TX did not induce protective effects in REST cKO mice. Red arrows (↓↑) Mn effects; blue arrows (↑↓) TX attenuated Mn effects; blue arrows (→) TX did not attenuate Mn effects. **(B)** Overview of TX’s neuroprotective effects against Mn toxicity, illustrating both REST-dependent and REST-independent pathways.

Our findings demonstrate for the first time that TX affords protection against Mn-induced neurotoxicity in dopaminergic REST-deleted (cKO) female mice (Figure 11). TX attenuated several Mn-induced motor and cognitive impairments, as well as dopaminergic neuronal damage in these mice, but not to the degree of its protection in WT (Pajarillo et al., 2018). At the molecular level, dopaminergic REST played a role in regulating several TX-responsive genes, linked to antioxidant defense and mitochondrial integrity, particularly CAT, SOD2, and OPA1. Furthermore, the TX-induced enrichment of H3K36me3, an epigenetic mark associated with transcriptional regulation, was found to be dopaminergic REST-dependent. These results suggest that TX mediates neuroprotection through both dopaminergic REST-dependent and independent pathways (Figure 11B), warranting further studies to delineate the precise contribution of these pathways to TX’s protective effects.

Consistent with our findings in WT mice (Pajarillo et al., 2018) and evidence that dopaminergic REST deletion exacerbated Mn-induced motor deficits (Pajarillo et al., 2024), TX also mitigated Mn-induced impairments in motor coordination and recognition memory in both WT and REST cKO mice. Given that protection was not complete in REST cKO, particularly on total distance traveled, dopaminergic REST is likely contributing to TX’s protective effects against Mn-induced behavioral deficits. To investigate sex-specific effects, we evaluated TX’s protective efficacy against Mn toxicity in both WT and REST cKO male mice (manuscript under review). Notably, the protective effects in male mice closely mirrored those observed in females, with only minor differences at the molecular and biochemical levels. These findings suggest that sex-specific factors do not significantly influence TX’s protection against Mn-induced toxicity linked to dopaminergic REST.

At the molecular levels, TX attenuated Mn-induced dopaminergic neuronal injury and the reduction of TH expression in REST cKO mice, paralleling its previously reported protective effects in the SN of WT female mice (Pajarillo et al., 2018), and its mitigation of Mn-induced dopamine loss in WT male rats (Owoyemi et al., 2024). The findings that TX attenuated the Mn-reduced TH levels even without dopaminergic REST suggest that dopaminergic REST is not essential for TX’s modulation of TH, possibly implicating alternative mechanisms. Although REST has been shown to upregulate TH expression in dopaminergic cultures and in mice (Pajarillo et al., 2024; Pajarillo et al., 2020b), the TX-increased REST expression in the striatum of REST cKO mice (Figure 3D) likely originates from non-dopaminergic neurons and/or glial cells. One potential mechanism is the TX-mediated activation of Wnt signaling, which, in turn, promotes REST expression in neuronal cultures (Digman et al., 2025). TX also attenuated Mn-induced upregulation of proapoptotic proteins (Figure 4), supporting its role in promoting dopaminergic neuronal survival (Pajarillo et al., 2018) and aligning with known neuroprotective functions of antioxidant genes (Lee et al., 2009; Wakade et al., 2008; Yazgan and Naziroglu, 2017). TX appears to induce a higher protection against Mn-increased Bax expression in the midbrain, compared to the striatum of REST cKO mice. Although the different effects of TX on Bax in these two regions are not fully understood, dopaminergic REST may be more critical in TX’s protective effect against Mn-induced proapoptotic Bax expression in the striatum, but it can effectively reduce Bax expression in the midbrain without dopaminergic REST, warranting further investigation.

Mn-induced apoptosis likely arises from oxidative stress and inflammation (Bahar et al., 2017). Among the antioxidant genes examined, TX failed to attenuate Mn-induced dysregulation of CAT, SOD2, and HO-1/2 in REST cKO mice, unlike its efficacy in WT mice, suggesting that dopaminergic REST might be required for TX to mitigate oxidative stress through these pathways.

Given that TX afforded some level of neuroprotection even in the absence of dopaminergic REST, it is plausible that its effects are mediated through alternative mechanisms involving non-dopaminergic REST-expressing cells, such as non-dopaminergic neurons, or glial cells, including astrocytes and microglia. Microglia, the primary source of proinflammatory cytokines in the CNS, and astrocytes, which regulate glutamatergic excitotoxicity via glutamate transporters, likely contribute to TX’s protective effects in REST cKO mice. This is supported by our findings that TX attenuated Mn-induced elevations of IL-1β and TNF-α and Mn’s downregulation of astrocytic glutamate transporters GLAST and GLT-1 in REST cKO mice (Figures 6, 7). Accordingly, TX’s neuroprotective effects may be mediated, at least in part, via its attenuation of Mn-induced inflammation and excitotoxicity, potentially involving astrocytes and microglia in the absence of dopaminergic REST.

Epigenetic modifications, such as histone acetylation and methylation, play critical roles in regulating transcriptional responses to Mn-induced neurotoxicity (Guo et al., 2018; Yang et al., 2022). Histone acetylation, regulated by HATs, promotes gene expression by loosening chromatin structure (Legube and Trouche, 2003). Our data show that TX restored Mn-suppressed H3K27 acetylation, a key epigenetic mark of active transcription. This restoration may underlie TX’s ability to mitigate Mn-induced repression of antioxidant genes, such as CAT and SOD2, which are crucial for oxidative stress defense (Yang et al., 2022). Notably, TX failed to restore the expression of these genes in REST cKO mice (Figure 5), indicating that dopaminergic REST is necessary for the modulation of their transcription by TX. However, PRDX1/5 was an exception, suggesting gene-specific dependency on REST.

TX also attenuated the Mn-induced reduction in protein levels of CBP/p300, a HAT protein that acetylates histone proteins, including histone H3 residues, such as H3K27, thereby enhancing chromatin accessibility to TFs (Tie et al., 2014). This effect was observed in both WT and REST cKO (Figures 8A,C), suggesting that TX attenuates Mn toxicity through CBP/p300-associated epigenetic regulation, and that dopaminergic REST is not critical for this TX-induced protective effect. However, further studies are required to fully understand the mechanistic role of CBP/p300 for TX’s neuroprotection.

Mn decreased levels of H3K36me3, a histone mark linked to active transcription (Kolasinska-Zwierz et al., 2009), an effect attenuated by TX in WT mice but not REST cKO mice, suggesting that dopaminergic REST is critical for TX’s ability to attenuate Mn-reduced H3K36me3 levels. Although H3K36me3’s role in Mn toxicity remains unclear, it has been linked to oxidative damage in arsenic-exposed human populations (Ma et al., 2016), supporting our findings. On the other hand, TX attenuation of Mn-increased H3K9/14ac did not require dopaminergic REST, highlighting complex mechanisms for TX’s modulatory effects with dopaminergic REST on epigenetic modification. The role of H3K9ac in neurotoxicity remains unclear; however, studies have shown that it is sensitive to cellular stress. For instance, increased H3K9ac has been associated with lead-induced cytotoxicity and elevated amyloid-β in SH-SY5Y cells (Wang et al., 2019) as well as ischemia-induced cytokine expression in the mouse brain (Patnala et al., 2017).

Beyond histone modifications, TX modulated H3K27ac enrichment at specific gene promoters in response to Mn, parallel with transcriptional changes in TH, REST, and CAT. For example, TX attenuated Mn-decreased H3K27ac enrichment at the TH promoter similarly in both genotypes (Figure 9A), suggesting dopaminergic REST-independent effects on TH regulation. However, overall H3K27ac binding at the TH promoter was lower in REST cKO compared to WT, consistent with prior evidence that REST positively regulates TH transcription (Pajarillo et al., 2024; Pajarillo et al., 2020b).

TX also restored H3K27ac enrichment at the REST promoter in both genotypes, though the effect was diminished in REST cKO mice. Since the REST promoter remains intact in REST cKO mice (which lack exon 4), transcriptional regulation can still occur at this region (Gao et al., 2011). Nevertheless, dopaminergic REST appears necessary for full TX-mediated recovery of REST expression. Given the absence of detectable REST protein in the midbrains of REST cKO (Pajarillo et al., 2024), TX’s ability to attenuate Mn-reduced REST protein expression in the midbrain may be via other non-dopaminergic neuronal cells.

The binding of REST to the TH promoter was markedly reduced in REST cKO compared to that in WT mice (Figure 9C), confirming that dopaminergic REST directly regulates TH transcription *in vivo*. On the other hand, TX’s ability to attenuate Mn-dysregulated H3K27ac interactions at the promoters of CAT, SOD2, and OPA1 was observed only in WT mice, not in REST cKO mice, paralleling mRNA changes (Figure 5), indicating that dopaminergic REST might be required for TX-mediated transcriptional regulation of these genes via histone acetylation.

Modulation of ER expression and signaling may also contribute to TX-mediated neuroprotection, as supported by the previous studies that SERMs, including TX and raloxifene, exerted protective effects in models of cerebral ischemia and spinal cord injury via the ER pathways (Nakamura et al., 2006; Hu et al., 2012; Carswell et al., 2004; Elzer et al., 2010). TX may also exert protection against Mn toxicity through ER-independent mechanisms, such as antioxidant activity (Zhang et al., 2007). Our findings show that Mn decreased ER-α and ER-β protein levels in the nigrostriatal region, while TX attenuated these reductions in both genotypes (Figure 10), suggesting no critical contribution of dopaminergic REST to TX effects on ERs.

Using DAT-Cre mice to delete dopaminergic REST in REST cKO mice may have off-target effects in non-dopaminergic subpopulations, as DAT-Cre activity has been reported in subsets of glutamatergic and GABAergic neurons in the lateral septum, amygdala, and lateral habenula (Papathanou et al., 2019). In our study, we did not observe phenotype differences between DAT-Cre, REST cKO, and REST loxP control mice, suggesting that the DAT-Cre impacts on non-dopaminergic subpopulations were minimal. Nevertheless, the potential off-target effects of DAT-Cre may need to be considered when interpreting these results.

Taken together, our findings suggest that dopaminergic REST might be required for the full neuroprotective efficacy of TX against Mn toxicity. TX-mediated attenuation of Mn-repressed antioxidant (CAT, SOD2, and HO-1/2) and mitochondrial (OPA1) genes requires dopaminergic REST (Figure 11). Nonetheless, TX retains significant neuroprotective capacity in the absence of dopaminergic REST, likely through dopaminergic REST-independent mechanisms involving non-dopaminergic neurons and glial cells, warranting further investigation into its mechanisms of action, particularly at the epigenetic and cell-type-specific levels.

## Data Availability

The raw data supporting the conclusions of this article will be made available by the authors, without undue reservation.
